# Inhibitory Effect of a Rosmarinic Acid-Enriched Fraction Prepared from Nga-Mon (*Perilla frutescens*) Seed Meal on Osteoclastogenesis through the RANK Signaling Pathway

**DOI:** 10.3390/antiox10020307

**Published:** 2021-02-17

**Authors:** Kanokkarn Phromnoi, Maitree Suttajit, Chalermpong Saenjum, Pornngarm Limtrakul (Dejkriengkraikul)

**Affiliations:** 1 Division of Biochemistry, School of Medical Sciences, University of Phayao, Phayao 56000, Thailand; kanokkarn.ph@up.ac.th (K.P.); maitree.suttajit@gmail.com (M.S.); 2 Department of Pharmaceutical Sciences, Faculty of Pharmacy, Chiang Mai University, Chiang Mai 50200, Thailand; 3 Cluster of Excellence on Biodiversity-Based Economics and Society (B.BES-CMU), Chiang Mai University, Chiang Mai 50200, Thailand; 4 Department of Biochemistry, Faculty of Medicine, Chiang Mai University, Chiang Mai 50200, Thailand; 5 Center for Research and Development of Natural Products for Health, Chiang Mai University, Chiang Mai 50200, Thailand

**Keywords:** Nga-Mon (*Perilla frutescens*), osteoclastogenesis, antioxidant, anti-inflammation, ROS, RANKL

## Abstract

The aim of this study is to determine antioxidant and anti-inflammatory activities relating to the antiosteoporosis effects of various perilla seed meal (PSM) fractions. The remaining waste of perilla seed obtained from cold oil compression was extracted with 70% ethanol and sequentially fractionated according to solvent polarity with hexane, dichloromethane, ethyl acetate, and water. The results indicated that the seed-meal ethyl acetate fraction (SMEF) exhibited the highest antioxidant and anti-inflammatory activities, and rosmarinic acid (RA) content. The signaling pathways induced by the receptor activator of the nuclear factor kappa B (NF-κB) ligand (RANKL) that trigger reactive oxygen species (ROS) and several transcription factors, leading to the induction of osteoclastogenesis, were also investigated. The SMEF clearly showed attenuated RANKL-induced tartrate-resistant acid phosphatase (TRAP)-positive multinucleated osteoclasts and TRAP activity. A Western blot analysis showed that the SMEF significantly downregulated RANKL-induced NF-κB, AP-1 activation, and the nuclear factor of activated T-cell 1 (NFATc1) expression. SMEF also suppressed RANKL-induced osteoclast-specific marker gene-like MMP-9 using zymography. Furthermore, the SMEF showed inhibition of RANKL-induced ROS production in RAW 264.7 cells. The results suggest that the SMEF, which contained high quantities of RA, could be developed as a natural active pharmaceutical ingredient for osteoclastogenic protection and health promotion.

## 1. Introduction

Bone homeostasis is associated with the balance between osteoclast-mediated bone resorption and osteoblast-induced bone formation. Impaired osteogenesis and excessive osteoclastogenesis can cause disorders such as Paget’s disease, rheumatoid arthritis, metastatic cancers, and osteoporosis [1]. Osteoporosis is a highly prevalent disease that has become a major public health problem [2] and is considered to be associated with chronic low-grade systemic inflammation and oxidative damage [3,4,5]. Bone inflammation induces the production of pro-osteoclastogenic cytokines; namely, the receptor activator of nuclear factor kappa-Β ligand (RANKL), tumor necrosis factor-α (TNF-α), and interleukins (IL). Moreover, it can induce the production of oxidative stress such as reactive oxygen species (ROS) and reactive nitrogen species (RNS), which can alter the bone-remodeling process, and make bones weak and fractured [6]. Osteoclasts are multinucleated cells that are differentiated from monocyte–macrophage lineage precursor cells, and are mainly triggered by two hematopoietic factors; namely, macrophage colony-stimulating factor (M-CSF) and RANKL [7,8].

The binding of RANKL to its receptor, RANK, triggers the activation of TNF receptor-associated factors, followed by the activation of multiple downstream signaling pathways, including NF-κB, AP-1 (c-Jun and c-Fos), mitogen-activated protein kinase (MAPK), and the Src/phosphatidylinositide (PI) 3-kinase/Akt axis, which subsequently induces the activation of critical transcription factors NFATc1 for the regulation of genes involved in the osteoclast differentiation and bone-resorption activities of mature osteoclasts, including tartrate-resistant acid phosphatase (TRAP), cathepsin K (CTSK), and matrix metalloproteinase-9 (MMP-9) [9,10]. Thus, agents suppressing RANKL signaling may inhibit osteoclastogenesis and bone loss.

Antiosteoporotic drugs such as bisphosphonates, calcitonin, estrogen, and selective estrogen receptor modulators (SERMs) can reduce vertebral fractures, ranging from 44% to 75% in patients with no pre-existing fractures, and from 30% to 47% in patients with pre-existing fractures [9]. However, these synthetic drugs can cause adverse side effects [10,11]. Therefore, it would be interesting to design natural compounds with antioxidant and anti-inflammatory activities for the attenuation of osteoclast differentiation.

*Perilla frutescens* L. (perilla or Nga-Mon in Thai) is an herb that belongs to the mint family, traditionally grown in northern Thailand. Previous studies showed that the Thai perilla-leaf extract is rich in polyphenols and flavonoids such as rosmarinic acid (RA), luteolin, and apigenin. This extract can inhibit oxidative stress, such as the production of ROS and NO, and decrease the production of proinflammatory cytokines (IL-1, IL-6, iNOS, and COX-2) in RAW264.7 cells [12]. Furthermore, previous in vitro and in vivo studies found that perilla extract can inhibit breast cancer [13], colon cancer [14], and gastric ulcers [15].

Perilla seeds are considered to be a rich source of essential fatty acids, including omega-3 and omega-6 [16,17]. Suttajit et. al. reported that the composition of lipids and fatty acids in Thai perilla seeds significantly varied among samples collected from different locations [18,19]. In addition to fatty acids, perilla seed is also a rich source of polyphenolic compounds such as RA, apigenin, and luteolin (which exhibits antioxidants), and possesses anti-inflammatory, antimicrobial, antialdoreductase, and alpha-glucosidase-inhibitory activities [20,21,22,23,24]. Commercially, perilla seed and its products are used in the health-food industry.

In agriculture, the perilla seed oil industry generates waste that is mostly discarded or used as low-value animal feed. Several reviewed articles revealed that perilla seed meal (PSM) is also a source of RA and has many beneficial properties [25,26,27,28,29].

However, the effect of RA-rich fractions of seed meal on osteoclastogenesis through RANKL signaling pathways has never been explored and reported. We investigate the antioxidative and anti-inflammatory activities of PSM extract and its effect on RANKL-induced osteoclastogenesis in vitro.

## 2. Materials and Methods

### 2.1. Reagents and Chemicals

Recombinant mouse soluble RANKL was obtained from R&D Systems (Minneapolis, MN, USA). Dulbecco’s Modified Eagle Medium (DMEM) and α-Modified Eagle’s Medium (α-MEM) were purchased from Invitrogen (Carlsbad, CA, USA), while fetal bovine serum (FBS) and penicillin–streptomycin were acquired from Thermo Fisher Scientific (Burlington, ON, Canada). The tartrate-resistant acid phosphatase (TRAP) staining kit, RA, luteolin, and apigenin were obtained from Sigma-Aldrich (St. Louis, MO, USA). Specific antibodies against phospho-p65 (Ser536), p65, IκBα, c-Jun, NFATc1, PARP, β-actin, and goat anti-rabbit IgG-HRP secondary antibodies were purchased from Cell Signaling Technology (Danvers, MA, USA). Caffeic acid, rutin, quercetin, and kaempferol were purchased from Tokyo Chemical Industry Co., Ltd. (Tokyo, Japan).

### 2.2. Extract and Fraction Collection and Preparation

Perilla seed was collected from the Wiang-Sa district, Nan province, Thailand. The voucher specimen was prepared by Dr. Komsak Pintha and Dr. Payungsak Tantipaiboonwong with the code QSBG-K2, and certified by the Queen Sirikit Botanic Garden Herbarium, Chiang Mai, Thailand. Seed oil was extracted by a cold-press oil machine, and the byproduct (the seed meal) was collected for our experiments. Dried PSM (500 g) was extracted with 70% ethanol (EtOH) to obtain the crude EtOH extract, which was then sequentially partitioned with hexane (Hex), dichloromethane (DCM), and ethyl acetate (EtOAc), and the residue aqueous phase was water (H_2_O). Each fraction had the solvents removed using a rotary evaporator and lyophilizer. The obtained dried fractions were stored at 20 °C and suspended in dimethyl sulfoxide (DMSO) before use.

### 2.3. 2,2-Diphenyl-1-picrylhydrazyl (DPPH) Radical-Scavenging Assay

The DPPH free-radical-scavenging assay was determined as previously described [30]. Of the samples, 20 mL in various concentrations was mixed with 180 mL of freshly prepared DPPH methanolic solution and kept in the dark for 30 min. Then, absorbance was measured at 540 nm. Ascorbic acid was used as a positive control. Results are expressed as 50% DPPH decolorization (IC_50_).

### 2.4. 2,2-Azino-bis-3-ethylbenzthiazoline-6-sulfonic Acid (ABTS) Radical-Scavenging Assay

The ABTS free-radical-scavenging assay was performed as previously described [31] with slight modifications. The ABST solution was diluted in potassium persulfate and kept in the dark for 12–14 h. Before use, this solution was diluted with deionized water to give absorbance at 734 nm of approximately 0.70. Then, 10 mL of the samples in various concentrations was mixed with 990 mL of working diluted ABTS and incubated in the dark for 6 min; the decrease in absorbance was measured at 734 nm. The positive control was ascorbic acid. Results are expressed as 50% ABTS decolorization (IC_50_).

### 2.5. Superoxide Anion (O_2_^•−^)Radical-Scavenging Assay

The superoxide anion-scavenging method was based on the power of samples to inhibit formazan formation in a phenazine methosulfate (PMS)–β-nicotinamide adenine dinucleotide (NADH) system, and then analyzed by the reduction in nitro blue tetrazolium (NBT) [32]. The reaction mixture was mixed with different concentrations of the samples or positive controls (ascorbic acid and RA). PMS was added to initiate the reaction and incubated in the dark for 5 min; absorbance was measured at 560 nm. Decreased absorbance indicated increased superoxide-anion-scavenging activity.

### 2.6. Nitric Oxide (NO) Radical-Scavenging Assay

An in vitro Griess colorimetric assay was used to determine NO-scavenging activity. The reaction mixture consisted of sodium nitroprusside solution, and samples were incubated at 37 °C for 150 min and transferred to a 96-well plate. The Griess reagent was added and incubated at room temperature for 5 min; absorbance was measured at 540 nm [12].

### 2.7. Ferric Reducing/Antioxidant Power (FRAP) Assay

The FRAP assay was performed as previously described [12]. The FRAP reagent was mixed with each sample and incubated at 37 °C for 4 min. Absorbance was measured at 593 nm. Standard solutions consisted of FeSO_4_·7H_2_O at different concentrations. The positive control was ascorbic acid. Results are expressed as milligrams of Fe(II) per 1 g of fraction (mg FeE/g fraction).

### 2.8. Total Phenolic Content (TPC)

TPC was determined using the Folin–Ciocalteu method [30]. Briefly, 20 μL of the sample was mixed with 100 μL of 10% Folin–Ciocalteu reagent and 80 μL of 7.5% Na_2_CO_3,_ and incubated at room temperature for 30 min. After that, the developed color was measured at 765 nm. TPC was calculated using a standard curve of gallic acid and expressed as milligrams of gallicacid equivalent per 1 g of fraction (mg GAE/g fraction).

### 2.9. Total Flavonoid Content (TFC)

TFC was examined using the aluminum chloride colorimetric method [30]. Initially, 25 μL of the sample and 125 μL deionized water were mixed with 7.5 μL of 5% NaNO_2_ solution and incubated at room temperature for 6 min. Then, 15 μL of 10% AlCl_3_ was added and incubated for another 6 min. Color development was performed by adding 50 μL of 1 M NaOH. The final volume of the reaction mixture was adjusted to 250 μL using deionized water. The developed color was measured at 510 nm. TFC was estimated using a standard curve of catechin and expressed as milligrams of catechin equivalent per 1 g of fraction (mg CE/g fraction).

### 2.10. RA Identification

RA was determined by reverse-phase HPLC using an Agilent 1200 equipped with multiwavelength and fluorescence detectors, as previously described [12] with some modifications. Content peaks were detected using a UV detector at 325 and 350 nm. In the HPLC chromatogram, the peak area and retention time of the fraction were compared with phenolic standards (caffeic acid, rutin, RA, luteolin, quercetin, apigenin, and kaempferol). The amounts of RA in the samples were calculated and expressed as mg/g fraction. RA content was rechecked with a fluorescence detector with wavelength excitation at 330 nm and wavelength emission at 400 nm. All samples were measured in triplicate.

### 2.11. Cell Culture and Cytotoxicity Assay

Mouse macrophage cell line RAW 264.7 was obtained from American Type Culture Collection (Manassas, VA, USA). RAW 264.7 cells were cultured in DMEM containing 10% heat-inactivated FBS, 100 U/mL penicillin–streptomycin under 5% CO_2_ at 37 °C. For the viability assay, cells (1 × 10^4^ cells) were seeded into each well of a 96-well plate, and incubated with and without the sample at different concentrations (0–200 mg/mL) for 48 h. Then, 3-(4,5-dimethylthiazol-2yl)-2,5-diphenyltetrazolium bromide (MTT) was added and incubated at 37 °C for 4 h. The excess MTT dye solution was removed, and only MTT formazan that stained living cells was redissolved in DMSO. Color intensity was measured at 540 nm using a microplate reader. The cell viability of the tested fractions was calculated compared to the negative control.

### 2.12. Proinflammatory Protein (NO, iNOS, and COX-2) Production

The inhibitory effect of PSM fractions on LPS and IFN-γ-stimulated production of NO, iNOS, and COX-2 was investigated. Briefly, RAW 264.7 cells were seeded in a 96-well plate, treated with various concentrations of samples, and incubated for 12 h. Then, combined LPS and IFN-γ were added and incubated for 48 h, while culture supernatants were collected to measure NO production using Griess reaction, and cell lysates were measured for iNOS and COX-2 using an immunoassay kit. Results are represented as 50% inhibitory concentration values (IC_50_). RA and curcumin were used as a positive control.

### 2.13. Tartrate-Resistant Acid Phosphatase (TRAP) Staining

The in vitro osteoclast differentiation of the sample was measured as previously defined [33] with some modifications. RAW 264.7 cells (5.0 × 10^3^ cells/well) were seeded into a 96-well plate with DMEM culture medium supplemented with 10% FBS and 1% penicillin–streptomycin for 24 h. Then, the medium was replaced with a differentiation medium (α-MEM) containing 100 ng/mL RANKL and various concentrations of the sample, and the medium was changed every 3 days until Day 6. Cells were fixed with 4% paraformaldehyde for 5 min and washed with warm distilled water according to the manufacturer’s instructions for the leucocyte acid phosphatase kit (Sigma Kit No.387). TRAP-positive osteoclasts with more than 3 nuclei were considered to be osteoclasts and visualized under a microscope.

### 2.14. TRAP Activity Assay

After treatment with the sample for 6 days, the RAW264.7 cells were lysed with 0.1% Triton X-100 and incubated for 2 h in –80 °C. The lysate was defrosted at 37 °C. Subsequently, 25 µL of the substrate solution (1 mg/mL *p*-nitrophenyl phosphate; PNPP) was added to 25 µL of the lysate and incubated for 4 h at 37 °C. Then, 50 µL of 0.5 N NaOH was added. Color intensity was measured at 405 nm. *p*-Nitrophenol (PNP) was used as a standard. RA was used as the antiosteoclastogenesis control.

### 2.15. Western Blot Analysis

To investigate whether SMEF modulates RANKL-induced NF-κB and AP-1 activation in RAW 264.7 cells, cells (1 × 10^6^ cells/well) were seeded in a 12-well plate, pretreated with SMEF (50 µg/mL) for 12 h, and then exposed to RANKL for 0, 10, 30, and 60 min. Furthermore, we studied the dose-dependent manner of SMEF on RANKL-induced NF-κB, AP-1, and NFATc1 expression; cells were pretreated with various concentrations of SMEF (0–50 µg/mL) for 12 h and stimulated with RANKL (100 ng/mL) by using a suitable time point from the earlier experiment. Whole-cell, nuclear, and cytoplasmic extracts were prepared as previously described [34]. Extracts were separated by SDS-polyacrylamide gel electrophoresis and transferred to a nitrocellulose membrane. The membranes were blocked with 5% BSA, probed with primary antibodies, and incubated at 4 °C overnight. Horseradish-peroxidase-conjugated secondary antibodies were then added. After incubation, the targeted proteins were visualized by enhanced chemiluminescence. Images were detected using an Omega Lum W Imager (Gel Company, Inc.) or exposed to the X-ray film (GE Healthcare Ltd., U.K.). β-Actin and PARP were used as the internal reference.

### 2.16. Gelatin Zymography

The secretions of MMP-9 from the cells were analyzed by gelatin zymography as previously described [34] with slight modifications. The culture supernatant of the RAW264.7 cells treated with the SMEF was collected and separated by 10% polyacrylamide gels containing 0.1% *w/v* of gelatin in nonreducing conditions. After electrophoresis, gels were soaked twice for 30 min in 2.5% *v/v* Triton X-100 to remove sodium dodecyl sulfate (SDS) to allow for the renaturation of MMPs, then they were incubated at 37 °C and kept for 48 h in an activating buffer containing 50 mM Tris-HCl, 200 mM NaCl, and 10 mM CaCl_2_ at pH 7.4. Bands were stained with 0.1% *w/v* Coomassie Brilliant Blue R and destained for 2 h at room temperature in a solution containing 10% acetic acid in 30% methanol. MMP-9 activity appeared as a clear band against a blue background. Images were taken using a Bio-Rad Gel Doc XR system (Hercules, CA, USA).

### 2.17. Measurement of Intracellular ROS

The production of ROS was determined through the measurement of the oxidation of 2′,7′-dichlorodihydrofluorescein diacetate (H_2_DCF-DA) to fluorescent 2′,7′-dichlorofluorescein (DCF) as previously described [35] with minor modifications. RAW 264.7 cells were seeded in a 96-well plate and pretreated with the SMEF (50 µg/mL) or positive control, 50 µM N-acetyl cysteine (NAC), and 250 µM L-ascorbic acid (Vit C) for 24 h, followed by treatment with 100 ng/mL RANKL for 1 h to stimulate ROS production. Then, 40 µM DCFH-DA solution was added, and fluorescence was detected after 30 min. Green fluorescent intensity was measured by a fluorescent microplate reader at excitation and emission wavelengths of 480 and 525 nm, respectively.

### 2.18. Statistical Analysis

Data are presented as mean ± standard deviation (SD). Statistical analysis was determined using one-way analysis of variance. Significant differences at the levels of *p* < 0.01, 0.05, and 0.001 were determined by Tukey’s multiple-comparison test. Data correlation was obtained by Pearson’s correlation test using IBM SPSS Statistics 22 and 26. Excel software was used to plot the graphs.

## 3. Results

### 3.1. Yield and Antioxidant Activity of PSM Fractions

The percentage yield of seed meal ethanolic crude extract (SME) was 5.6% *w/w*. The crude extract was sequentially partitioned with Hex, DCM, EtOAc, and H_2_O to obtain SMHF, SMDF, SMEF, and SMWF, respectively. The results showed that the highest yield was observed in H_2_O (10.89 g), and a declining level was sequenced by Hex (3.2 g), EtOAc (0.56 g), and DCM (0.39 g). Each fraction produced different yields, which may be due to the presence of phytochemical compounds in the fractions [36].

Oxidative stress and antioxidants are associated with the bone remodeling process [3,36]; therefore, the antioxidant capacity of PSM fractions was investigated through free-radical-scavenging activity on DPPH, ABTS, O_2_^–^, and NO radicals. The 50% inhibition concentration (IC_50_) was calculated, and the results of in vitro antioxidant determination are shown in Table 1. The SMEF possessed the highest antioxidant activity in all in vitro assays. To confirm this activity, in a FRAP assay, which is a study of electron transfer, the SMEF also had the highest FRAP value.

### 3.2. Anti-Inflammatory Activity of PSM Fractions

The production of inflammatory cytokines is related to oxidative stress, and promotes osteoclastogenesis and bone resorption [37,38,39,40]. Moreover, several articles reported that LPS and IFN-γ are a crucial cytokine for the inflammation process via the activation of proinflammatory mediators such as NO, iNOS, and COX-2 [41,42]. Our studies found an inhibitory effect of PSM fractions on the LPS and IFN-γ-stimulated production of NO, iNOS, and COX-2 in RAW264.7 cells. The IC_50_ of each fraction on the inhibition effect of proinflammatory proteins is displayed in Table 2. The most active response was seen in the SMEF compared to the positive control (curcumin and RA).

### 3.3. TPC, TFC, and RA content of the PSM fractions

Recently, in vivo and in vitro studies supported roles for polyphenols and flavonoids in the prevention of oxidative stress, including osteoporosis and inflammatory bone diseases [43,44,45,46,47]. Therefore, we studied the TPC and TFC on PSM fractions. As demonstrated in Table 3, the SMEF showed the highest phenolic and flavonoid content.

### 3.4. Quantitative HPLC Analysis of RA Compound from PSM Fractions

**Figure 1:**
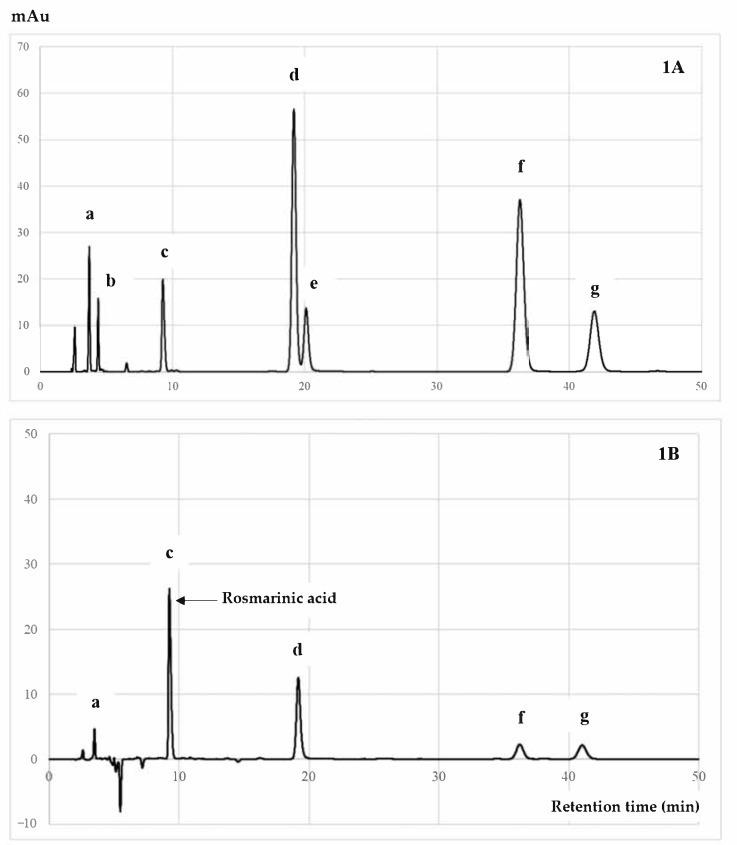
HPLC chromatogram of (**A**) mixed standards and (**B**) SMEF: (**a**) caffeic acid; (**b**) rutin; (**c**) rosmarinic acid; (**d**) luteolin; (**e**) quercetin; (**f**) apigenin; (**g**) kaempferol.

RA is an ester of caffeic acid and 3,4-dihydroxyphenyllactic acid that can be widely found in many plants, especially rosemary, perilla, basil, and mint [48]. RA possesses remarkable pharmacological activities, such as antioxidant and anti-inflammation properties, and with regard to neurodegeneration, cancer, diabetes, and bone diseases [48,49,50]. Our studies analyzed the RA level of each PSM fraction, with results showing that the SMEF contained the highest amount of RA, followed by SME, SMWF, SMHF, and SMDF, as shown in Table 3. Moreover, an HPLC chromatogram, as shown in Figure 1, indicated that RA was a major phytochemical compound isolated from the EtOAc fraction, followed by luteolin, apigenin, and kaempferol.

In our studies, the Pearson correlation test illustrated that the RA content of all analyzed fractions was significantly correlated to TPC, TFC, and reducing power activity (FRAP), with r^2^ of 0.986, 0.998, and 1.000; *p* < 0.01, respectively. Moreover, there was remarkable correlation between the RA level and antioxidant and anti-inflammatory activities. SMEF obtained the highest content of RA, which is related to its high antioxidant and anti-inflammatory activities, suggesting it is the active compound for inhibiting osteoclast differentiation through the RANK signaling pathway [27,51]. Thus, the effect of the RA-enriched fraction or SMEF on RANKL-induced osteoclast differentiation was also identified.

### 3.5. Cytotoxicity of SMEF in RAW 264.7 Cells

**Figure 2:**
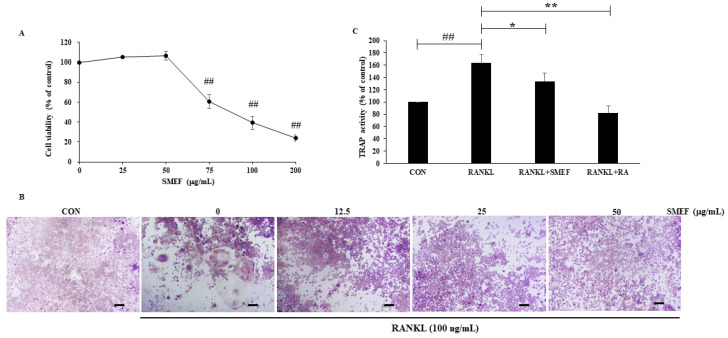
SMEF suppresses osteoclastogenesis in vitro. (**A**) RAW264.7 cells cultured with SMEF in concentration range of 0–200 μg/mL for 48 h in 96-well plates. Cell viability was determined by an MTT assay. ^##^ *p* < 0.001 compared with untreated control. (**B**) RAW264.7 cells were coincubated with SMEF (0–50 μg/mL) and RANKL (100 ng/mL) for 6 days and then stained using a leukocyte acid phosphatase (TRAP) kit. TRAP-positive multinucleated osteoclasts were visualized in 100× magnification under light microphotography. Scale bars, 100.2 μm. **(C)** RAW264.7 cells were co-incubated with SMEF (50 μg/mL) and RANKL (100 ng/mL) for 6 day and then measured using the TRAP solution assay. RA (100 μM) was used as a positive control and TRAP activity was expressed as % of control. The data is presented as the mean ± SD of three independent experiments. ^##^ *p* < 0.001, compared with control (CON); * *p* < 0.01, ** *p* < 0.001 compared with RANKL control.

The cytotoxic effect of SMEF (0–200 μg/mL) on RAW 264.7 cells was determined by MTT assay as shown in Figure 2A. There was no noticeable cytotoxicity in concentrations lower than 50 μg/mL. Therefore, we chose this SMEF concentration for further experiments.

### 3.6. Effect of SMEF on RANKL-Induced Osteoclast Differentiation

Osteoclast precursors are expressed using RANK receptors to differentiate into multinucleated TRAP-positive osteoclasts in response to the RANKL cytokine [52]. From the studies of Zeng X. Z. et al., and Kim J. et al. [53,54], the concentration of RANKL at 100 ng/mL can induce the osteoclastogenesis effect. Hence, in our studies, RAW 264.7 cells were incubated with RANKL (100 ng/mL) in the presence of SMEF (0–50 µg/mL) and allowed to differentiate into osteoclasts. TRAP activity was determined using a TRAP solution assay, having stained and visualized the multinucleated TRAP-positive cells by microscopy. Figure 2B demonstrates that RANKL induced osteoclast differentiation, and SMEF could impair such differentiation in a dose-dependent manner. Furthermore, we investigated whether the extract was effective on RANKL-stimulated TRAP activity in RAW 264.7 cells. RA was used as a positive control. The negative control (CON) was a nontreated group and set as 100%. TRAP activity increased by up to 163.4 ± 14.2% after treatment with RANKL compared with that of the control. SMEF significantly decreased RANKL-induced TRAP activity, similar to the positive control, as illustrated in Figure 2C.

### 3.7. Effect of SMEF on RANKL-Induced NF-κB and AP-1 Activation

**Figure 3:**
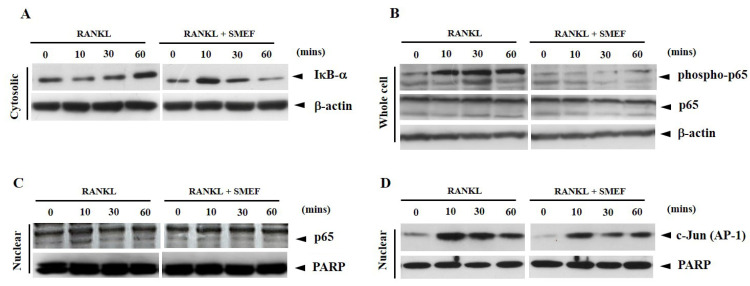
SMEF suppresses RANKL-induced NF-κB and AP-1 activation after inducing with RANKL for indicated time. (**A**–**D**) RAW 264.7 cells were pretreated with SMEF (50 μg/mL) for 12 h and then exposed to RANKL for 0, 10, 30, and 60 min. Cytosolic fractions were analyzed for protein content of IκB-α degradation, whole-cell lysates were used to determine phosphorylation levels of NF-κB p65, and nuclear fractions were analyzed for NF-κB p65 and AP-1(c-Jun) translocation (assayed by Western blotting as described in the Materials and Methods section).

The interaction of RANKL and RANK leading to the activation of NF-κB and AP-1 has an important role in osteoclastogenesis [55,56]. To determine the underlying SMEF inhibitory mechanism in osteoclast formation, a Western blot analysis of NF-κB (p65) and AP-1 (c-Jun) was used in our studies. Because the proteolytic degradation of IκB-α is required for the phosphorylation of NF-κB to the nucleus, we first examined IκB-α degradation in the cytoplasm after RANKL stimulation for various times. RANKL induced IκB-α degradation in control cells as early as within 10 min and returned to a normal level within 30 min (Figure 3A). On the other hand, cells pretreated with SMEF inhibited RANKL-induced degradation of IκB-α. We next examined whether SMEF affected NF-κB p65 phosphorylation and translocation. As shown in Figure 3B, RANKL started to activate NF-κB p65 phosphorylation within 10 min and until 60 min of treatment; however, SMEF abrogated RANKL-induced NF-κB phosphorylation. Following the phosphorylation results, RANKL could also induce NF-κB p65 translocation at 10 min, and SMEF decreased RANKL-induced NF-κB p65 translocation, especially at 10 min (Figure 3C). Therefore, SMEF could inhibit the RANKL-induced functional activities of NF-κB p65. To investigate whether SMEF modulated RANKL-induced AP-1 activation in RAW 264.7 cells, nuclear extracts were used to measure the translocation of AP-1 from the cytoplasm to the nucleus. As shown in Figure 3D, RANKL highly activated AP-1 (c-Jun) at 10 min, and SMEF inhibited RANKL-induced c-Jun expression in the nucleus of cells.

**Figure 4:**
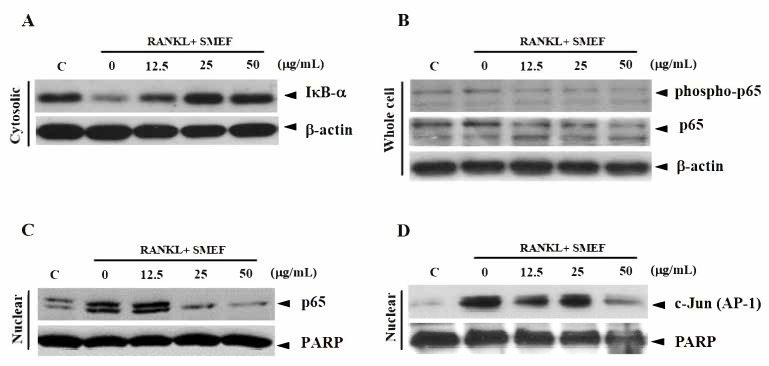
SMEF suppresses RANKL-induced NF-κB and AP-1 activation in dose-dependent manner. (**A**–**D**) RAW 264.7 cells were pretreated with SMEF (0, 12.5, 25, and 50 µg/mL) for 12 h and then exposed to RANKL for 10 min. Cytosolic fractions were analyzed for protein content of IκB-α degradation, whole-cell lysates were used to determine phosphorylation levels of NF-κB p65, and nuclear fractions were analyzed for NF-κB p65 and AP-1(c-Jun) translocation (assayed by Western blotting as described in the Materials and Methods section).

On the basis of the end-point bioassay for NF-κB and AP-1 activity, an exposure time of 10 min was suitable for RANKL-induced NF-κB and AP-1 activity. Next, we selected this time point (10 min) to study the dose-dependent manner of SMEF on RANKL-induced NF-κB and AP-1 expression. Cells were pretreated with various concentrations of SMEF (0–50 µg/mL) for 12 h and stimulated with RANKL (100 ng/mL) for 10 min. The results illustrated that RANKL significantly induced IκB-α degradation, NF-κB p65 phosphorylation, NF-κB p65 translocation, and AP-1 translocation, and treatment with SMEF impaired the RANKL-induced signaling molecules in a dose-dependent manner (Figure 4).

### 3.8. Effect of SMEF on Osteoclastic-Specific Protein Expression

**Figure 5:**
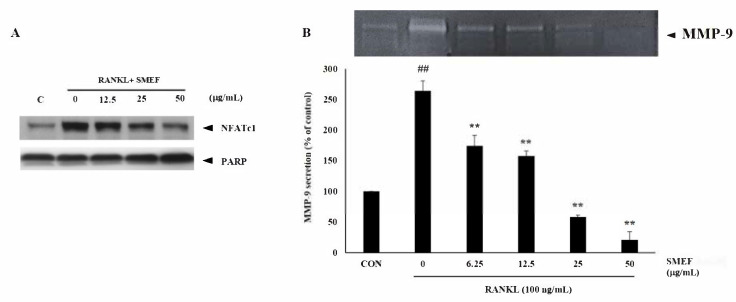
SMEF inhibits osteoclastic-specific proteins, NFATc1 and MMP-9 expression. (**A**) RAW264.7 cells were pretreated with SMEF in concentrations of 0, 12.5, 25, and 50 μg/mL for 12 h and stimulated by RANKL (100 ng/mL) for 10 min. Nuclear extracts were prepared and analyzed by Western blot analysis. (**B**) RAW264.7 cells were cotreated with SMEF in concentrations of 0, 12.5, 25, and 50 μg/mL and RANKL (100 ng/mL) for 2 days. Culture supernatants of treated cells were collected, and secretion of MMP-9 was analyzed by gelatin zymography. Data are presented as mean ± SD of three independent experiments. ^##^ *p* < 0.001 compared with control (CON); ** *p* < 0.001 compared with RANKL control.

Induction and activation of NFATc1 is important for RANKL-stimulated osteoclastogenesis [57,58]. Moreover, MMP-9 is the most abundant gelatinolytic enzyme in osteoclasts, and has a significant role in the invasive activity of osteoclasts [59]. To further identify the inhibitory effect of SMEF on RANKL-induced osteoclastogenesis, we investigated the expression of these osteoclast-specific proteins, NFATc1 and MMT-9, using Western blot and gelatin zymography assays, respectively. In response to RANKL, the expression of NFATc-1 was greatly upregulated after stimulation with RANKL for 10 min. However, in the presence of SMEF, the RANKL-dependent upregulation of NFATc-1 protein expression was substantially inhibited in a dose-dependent manner (Figure 5A).

Previous studies showed that RANKL can induce MMP-9 secretion after long-term incubation [60,61]. This is consistent with our study that showed MMP-9 activity being enhanced by up to 263.87 ± 16.2% after induction with 100 ng/mL RANKL for 2 days compared with that of the control. However, SMEF decreased RANKL-induced MMP-9 activity in a dose-dependent manner, as illustrated in Figure 5B.

### 3.9. Effect of SMEF on RANKL-Induced ROS Generation

**Figure 6:**
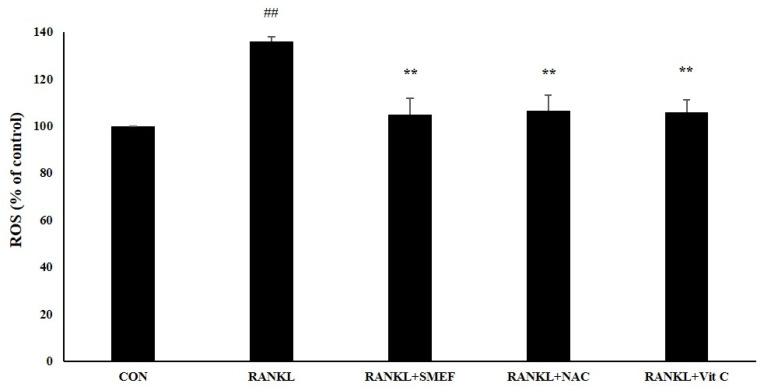
SMEF inhibits RANKL-induced reactive oxygen species (ROS) production using cellular dichlorodihydrofluorescein diacetate (DCF-DA) assay. RAW 264.7 cells were seeded in a 96-well plate, pretreated with the SMEF (50 µg/mL) for 24 h, and stimulated by RANKL (100 ng/mL) for 1 h. Then, 40 µM of DCFH-DA solution was added for 30 min. Treated cells were measured with a fluorescent microplate reader. Then, 50 µM N-acetylcysteine (NAC) and 250 µM ascorbic acid (Vit C) were used as a positive control. All data are presented as the mean ± SD of three independent experiments. ^##^ *p* < 0.001 compared with the untreated control (CON); ** *p* < 0.001 compared with RANKL treatment (RANKL).

RANKL induces intracellular ROS production, and ROS-activated osteoclast differentiation occurs [62]. We determined that the SMEF affected RANKL-induced ROS generation. ROS production was increased through RANKL stimulation by up to 135.9 ± 2.11% of the control. However, the generation of intracellular ROS was attenuated to the negative control by the treatment of SMEF (50 µg/mL), similar to NAC and vitamin C (Figure 6).

## 4. Discussion

Osteoporosis is a severe systemic bone-remodeling disease characterized by excessive bone resorption by osteoclasts, resulting in impaired bone mass and increased high fracture risk. Moreover, oxidative stress and proinflammatory cytokines can activate the differentiation of osteoclasts and osteoporosis. Many investigators reported that abundantly polyphenolic antioxidants such as genistein, quercitrin, ferulic acid, piceatannol, and taxifolin can inhibit the degradation of the bone matrix in osteoclasts, and induce alkaline phosphatase activity and matrix protein synthesis in osteoblasts [44,63,64,65,66]. Plants are the main sources of phytochemicals, antioxidants, and anti-inflammatory agents [3,30]. There was an association between the increased intake of fruit and vegetables and the decrease of bone fracture risk [67]. It is suggested that antioxidants and anti-inflammatory compounds could be used for osteoporosis prevention and treatment.

PSM is usually discarded or used in animal feeds as a low-value material. However, some studies [68,69] discovered that it still contained easily digestible proteins, polysaccharides, and substantial amounts of phenolic antioxidants, including RA. Our laboratory revealed that PSME clearly showed in vitro antimutagenicity, and antioxidant and anti-inflammatory activities [70]. RA, of which large quantities are found in various mint plants [71], including perilla [12,13,70], exhibits antioxidant, anti-inflammatory, antiallergic, and α-glucosidase-inhibitory activities [48,72,73,74,75]. Moreover, RA can inhibit osteoclast differentiation [27] and promote osteoblastic cells. The use of PSM in health promotion requires a scientific report and experiment-based data to explain the molecular mechanisms associated with preventive medicine in future applications.

Results indicated that the PSM EtOAc fraction exhibited the highest antioxidant (Table 1) and anti-inflammatory activities (Table 2), as well as polyphenols, flavonoids, and RA contents (Table 3 and Figure 1). The SMEF and so-called RA-enriched fraction were used to explore osteoclastogenesis effects. Ours is the first report showing the inhibitory effect of SMEF on osteoclastogenesis in vitro and in mouse macrophage RAW 264.7 cells. Our results indicated that SMEF potentially inhibited the RANKL-induced formation of multinucleated cells. SMEF suppresses the RANKL-induced activity of TRAP, which is an essential enzyme in osteoclast differentiation (Figure 2B,C). To investigate the molecular mechanisms associated with the inhibitory effects of SMEF, Western blotting was used. The results showed that RANKL activated NF-κB in osteoclast precursor cells through the degradation of IκB-α, and subsequent NF-κB phosphorylation and translocation. These results are correlated with those previously reported by Zhu M. et al. [52]. However, SMEF showed an inhibitory effect on RANKL-induced IκB-α degradation, leading to the suppression of NF-κB p65 phosphorylation and translocation (Figure 3 and Figure 4). Moreover, AP-1 (c-fos and c-Jun) plays a role in regulating osteoclastogenesis. The inhibition of AP-1 activity reduces osteoclast differentiation [76]. In this study, we investigated the translocation of c-Jun in RANKL-induced RAW 264.7 cells. Our results demonstrated that SMEF reduced RANKL-induced c-Jun translocation to the nucleus of RAW 264.7 cells (Figure 3 and Figure 4). These results suggested that SMEF could reduce osteoclastogenesis by the inhibition of NF-κB and AP-1 activation. Transcription factor NFATc1 is activated after NF-κB and AP-1 stimulation [7,77,78]. Then, osteoclast-specific genes and MMP-9 are expressed, eventually initiating osteoclastogenesis and bone resorption. Our results found that SMEF can abrogate RANKL-induced NFATc1 and MMP-9 expression (Figure 5). Therefore, SMEF, which is enriched with RA, inhibits the RANKL-induced NF-κB and AP-1 signaling pathway, and thereby contributes to the suppression of osteoclast formation. Intracellular ROS increased following RANKL stimulation [79]. Our study demonstrated that ROS in RAW 264.7 cells after RANKL stimulation were scavenged by SMEF treatment (Figure 6). Our results showed that SMEF at 50 µg/mL, which is a nontoxic dose from the MTT assay, had good effects on the inhibition of RANKL-mediated osteoclastogenesis, so this concentration may be safe for future use. However, our experiments on the biological properties of SMEF were only intensively performed by in vitro methods and cell-based studies. Therefore, future research should be more focused on in vivo and clinical studies. Their beneficial applications, and possible toxicity and safety issues, need to be warranted by such evidence.

## 5. Conclusions

This is the first study to demonstrate that the RA-enriched PSM fraction SMEF, which contains the highest antioxidant and anti-inflammatory polyphenols, can exhibit an inhibitory effect on osteoclast differentiation and its underlying mechanism in preosteoclastic RAW 264.7 cells. SMEF remarkedly suppressed RANKL-induced ROS production, downregulated the expression of osteoclast-associated genes (MMP-9), and significantly modulated the activation of transcriptional factors such as NF-κB, AP-1, and NFATc1. Waste-product PSM could be developed as a natural active pharmaceutical ingredient for osteoclastogenic protection and health promotion. However, its beneficial applications need to be confirmed by more in vivo and clinical evidence.

## Figures and Tables

**Table 1:** Antioxidant activity as IC_50_ and ferric reducing/antioxidant power (FRAP) of perilla seed meal (PSM) fractions determined by different assays using ascorbic acid and rosmarinic acid (RA) as the positive control.

Fractions and Standards	Radical Scavenging Assay IC_50_ (µg/mL)				Reducing Power Assay mg Fe/g Fraction
	DPPH^•^	ABTS^•+^	O_2_^•−^	NO	FRAP
SME	76.6 ± 5.1 ^b^	20.7 ± 0.4 ^c^	28.7 ± 1.39 ^c^	40.6 ± 1.32 ^c^	1058.2 ± 110.3 ^d^
SMHF	>200 ^d^	86.1 ± 5.9 ^e^	81.9 ± 3.25 ^d^	95.9 ± 3.04 ^e^	240.6 ± 15.0 ^e^
SMDF	162.9 ± 11.7 ^c^	16.2 ± 0.2 ^b^	92.8 ± 3.37 ^e^	100.1 ± 3.20 ^f^	460.4 ± 17.1 ^e^
SMEF	9.2 ± 0.7 ^a^	3.6 ± 0.1 ^a^	19.5 ± 1.13 ^b^	26.6 ± 0.87 ^b^	8627.3 ± 351.7 ^b^
SMWF	25.6 ± 1.4 ^a^	30.6 ± 0.5 ^d^	32.0 ± 1.79 ^c^	43.9 ± 1.24 ^d^	792.1 ± 31.7 ^d^
L-ascorbic acid	13.56 ± 1.29 ^b^	2.06 ± 0.03 ^a^	13.1 ± 0.47 ^b^	-	7948.6 ± 217.0 ^c^
RA	-	-	6.9 ± 0.26 ^a^	17.7 ± 1.02 ^a^	20,572.7 ± 181.9 ^a^

Values expressed as mean ± SD (*n* = 3). Means with different letters in the same column are significantly different (*p* < 0.05).

**Table 2:** Anti-inflammatory activity as IC_50_ of PSM fractions determined by a Griess reaction assay and ELISA using RA and curcumin as positive control.

Fractions and Standards	IC_50_ (µg/mL)		
	NO	iNOS	COX-2
SME	28.1 ± 1.6 ^d^	34.4 ± 1.5 ^d^	39.4 ± 1.7 ^d^
SMHF	43.0 ± 1.6 ^e^	38.3 ± 0.6 ^e^	42.6 ± 0.1 ^d^
SMDF	>50	>50	>50
SMEF	21.2 ± 1.4 ^c^	24.2 ± 2.7 ^c^	26.9 ± 2.4 ^c^
SMWF	29.7 ± 2.0 ^d^	42.2 ± 7.6 ^f^	49.6 ± 7.8 ^e^
RA	13.2 ± 0.6 ^b^	17.5 ± 2.3 ^b^	21.3 ± 2.8 ^b^
Curcumin	7.5 ± 0.7 ^a^	8.9 ± 5.6 ^a^	9.4 ± 5.7 ^a^

Values expressed as mean ± SD (*n* = 3). Means with different letters in the same column are significantly different (*p* < 0.05).

**Table 3:** Total phenolic content (TPC), total flavonoid content (TFC), and RA content of PSM fractions.

Fractions	TPC	TFC	RA
	mg GAE/g Fraction	mg CAE/g Fraction	mg/g Fraction
**SME**	64.6 ± 1.3 ^b^	68.3 ± 5.8 ^b^	23.7 ± 1.1 ^c^
**SMHF**	18.3 ± 3.7 ^a^	7.3 ± 0.7 ^a^	2.9 ± 0.5 ^a^
**SMDF**	67.4 ± 8.9 ^b^	18.4 ± 7.1^a^	1.4 ± 0.3 ^a^
**SMEF**	291.4 ± 6.0 ^c^	493.4 ± 15.9 ^c^	243.8 ± 3.2 ^d^
**SMWF**	57.4 ± 2.9 ^b^	57.3 ± 4.6 ^b^	11.4 ± 0.8 ^b^

Values expressed as mean ± SD (*n* = 3). Means with different letters in the same column are significantly different (*p* < 0.05).

## Data Availability

All data is contained within the article.
